# Lithium interphase enhancement for applications in lithium–sulphur batteries

**DOI:** 10.1080/14686996.2025.2593686

**Published:** 2025-11-24

**Authors:** Antonio De Marco, Morteza Rahmanipour, Gioele Pagot, Giampaolo Lacarbonara

**Affiliations:** a Department of Chemistry “Giacomo Ciamician”, University of Bologna, Bologna, Italy; b Section of Chemistry for Technology, Department of Industrial Engineering, University of Padova, Padova, Italy

**Keywords:** Lithium metal, surface protection, lithium–sulphur batteries, energy storage materials, solid electrolyte interphase

## Abstract

Stable lithium plating/stripping of metallic lithium anode is considered as the urgent challenge for the development of post-lithium-ion batteries including lithium–sulphur and lithium–air batteries. In this work, we report a new facile and cost-effective method to grow a protective layer on the surface of lithium metal through immersing the lithium surface in a nitrogen-saturated solution that eliminates the operational restrictions of reported modification approaches in controlled atmosphere. N_2_-treated lithium shows prolonged cycling in a symmetric configuration and chemical stability. We demonstrate that the treated Li anode notably enhances the cycling stability, coulombic efficiency, as well as the rate capability of lithium–sulphur cells.

## IMPACT STATEMENT

This work introduces a simple, low-cost nitrogen-treatment method enabling stable lithium plating/stripping in ambient conditions, forming a protective interphase that enhances cycling stability and efficiency in lithium–sulphur batteries.

## GRAPHICAL ABSTRACT

**Figure:**
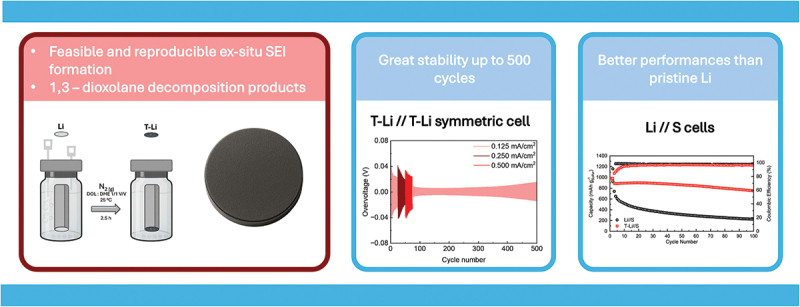

## 1. Introduction

Albeit lithium metal exhibits the lowest electrode standard potential (−3.04 V vs. standard hydrogen electrode) and high theoretical specific capacity (3860 Ah kg^−1^), the practical application of Li electrode is dominantly limited by its high reactive nature with the liquid electrolyte, huge volume change, and dendrite formation induced by inhomogeneous current density distribution during cycling [1,2].

Recent studies have provided a deeper mechanistic understanding of these degradation processes, elucidating how electrochemical, chemical, and mechanical factors collectively govern the nucleation, growth, and stripping behaviour of lithium during cycling [3]. The dynamic interplay between interfacial reactions, ion transport results in the progressive evolution of surface morphology, solid electrolyte interphase (SEI) heterogeneity, and the formation of inactive ‘dead Li’, ultimately leading to poor coulombic efficiency and limited reversibility. To overcome these issues, several strategies have been developed to stabilize lithium metal anodes, including electrolyte engineering, interfacial modification, and structural optimization [4]. Among them, surface and interface engineering has emerged as a particularly effective route, enabling the design of artificial SEI layers with tailored chemical composition, ionic conductivity, and mechanical strength [5]. Artificial protective coatings formed through chemical, electrochemical, or gas-phase treatments can suppress dendrite formation, accommodate Li volume changes, and mitigate parasitic side reactions at the Li–electrolyte interface [5]. Furthermore, the combination of advanced *in situ* and operando analytical techniques has substantially improved our understanding of Li plating/stripping mechanisms, allowing the direct observation of morphological evolution and interfacial transformations under realistic operating conditions [6]. These recent advances highlight the critical importance of interfacial design and mechanistic insight for achieving stable and reversible lithium metal electrodes. Among the *in-situ* approach, it is worth mentioning the tuning of the lithium metal interphase composition and morphology by utilizing different electrolyte additives including LiNO_3_, fluoroethylene carbonate (FEC), and halogenated compounds like HF and CsPF_6_ [7–12]. Electrolyte additives play incipient roles in establishing a more stable SEI between the metallic lithium anode and the electrolyte. The choice of the electrolyte additive strongly depends on its stability window that should be compatible with the operating voltage of the aimed battery. LiNO_3_ has been widely utilized as an electrolyte additive that forms a Li_2_O-rich SEI on lithium surface, and when lithium metal is used as the anode in lithium–sulphur (Li//S) battery, LiNO_3_ additive contributes effectively in both confining polysulfides at the cathode side and preventing the reduction of soluble polysulfides with the Li anode [13–15]. However, there is not a unanimous agreement in the literature about its concentration inside the electrolyte. The reason relies on the fact that LiNO_3_ is continuously consumed during the long-term battery operation. Rodriguez et al. reported the use of 1 M LiTFSI with 1 M LiNO_3_ in an ether-based solvent inhibits the 3D growth of lithium [16]. Shim et al. investigated the Li//S battery performance using electrolytes at various concentrations of LiNO_3_ and concluded the occurrence of irreversible reactions at excessive LiNO_3_ concentration [17]. The additive concentration of 0.8 M sounds an optimum concentration for symmetric cell, and it has been also adopted in other Li//S studies [18]. However, this concentration, corresponding to the maximum reported solubility of LiNO_3_ in our electrolyte, may cause unwanted irreversible reactions chiefly in the cathodic side which appears as an additional plateau in the discharge profile approximately within 1.8–1.9 V [17]. In order to benefit simultaneously from the positive effect of LiNO_3_ in achieving stable lithium stripping/plating and avoid its detrimental effect in the cathodic side, our approach was to use a lower additive concentration of 0.45 M.

SEI enhancement can also be achieved through *ex-situ* fabrication of artificial SEI onto the lithium surface prior to the cell assembly [19]. Artificial SEIs are typically inorganic protective layers that are grown on the substrate in a high-precision, tuneable manner [20]. As a result, compared to SEIs formed through the electrolyte additive method, artificial SEIs are more conformal, exhibiting better chemical and mechanical stability [21]. It is extensively reported that the parameters associated with battery operation, including rate capability, cycling performance, and formation of dendrites strongly depend on the quality of the grown protective layer [22–25]. Ideally, an artificial SEI must be a highly electronic insulator, a highly lithium-ion conductor, chemically and mechanically stable, and sufficiently thick without dead zones [26,27]. Lithium metal readily reacts with gaseous nitrogen even at room temperature, forming lithium nitride (Li_3_N), which can serve as an SEI layer [28]. Li_3_N is highly reactive due to its dual nature as a superbase and a strong nucleophile. This reactivity enables its use as a precursor in ammonia synthesis under mild conditions [29]. In addition, Li_3_N functions effectively as a nucleophile in C–N coupling reactions, driven by the high nucleophilicity of the nitride ion (N^3−^). It reacts with electrophilic carbon species, such as acyl chlorides or aryl halides to form C–N bonds. For instance, the reaction between Li_3_N and acyl chlorides yields aryl imides via two successive nucleophilic attacks. These properties make Li_3_N a valuable reagent for the synthesis of nitrogen-containing organic compounds, particularly in transformations requiring strong nucleophiles [30].

1,3-Dioxolane (DOL) is a widely utilized solvent in lithium–sulphur (Li–S) battery systems due to its favourable electrochemical properties and ability to dissolve lithium salts [31]. Its five-membered cyclic acetal structure enables three distinct types of ring-opening reactions, each yielding different products depending on the reaction conditions. The cationic ring-opening polymerization (CROP) of DOL, typically initiated by a Lewis acid, leads to the formation of poly(1,3-dioxolane) (pDOL) along with various side products. This process has been extensively studied, particularly for applications in polymer electrolytes [32]. In addition to CROP, radical polymerization of DOL has emerged as an alternative route for synthesizing pDOL. Remarkably, this method has been employed to prepare composite polymer electrolytes without the need for an initiator or crosslinking agent, offering a simplified synthetic pathway and potential advantages in processability and purity [33]. The anionic ring-opening of DOL remains the least explored. This pathway can be triggered in the presence of strong nucleophiles, taking advantage of the moderately electrophilic character of the methylene carbon flanked by the two oxygen atoms. As suggested by Aurbach and co-workers, such reactivity may be relevant in electrolyte degradation processes or in the design of functionalized materials [33].

Herein, we introduce an economical reproducible method to form a stable and uniform thin layer of DOL ring opening reaction products on the Li surface by debottlenecking the existing approaches that are primarily limited to gas-phase treatment and dry rooms. Composition of the lithium–electrolyte interphase has been elucidated via electrochemical and chemical–physical techniques, such as electrochemical impedance spectroscopy (EIS), X-ray diffraction (XRD), X-ray photoelectron spectroscopy (XPS), and Fourier-transformed infrared spectroscopy-attenuated total reflectance (FTIR-ATR). The stripping/plating behaviour of the treated lithium has been studied in the symmetric configuration and the viability of the treated-Li electrode (T-Li) as a promising anode is demonstrated in Li//S battery.

## 2. Experimental section

### 2.1. Preparation of protected lithium

**Figure 1.**
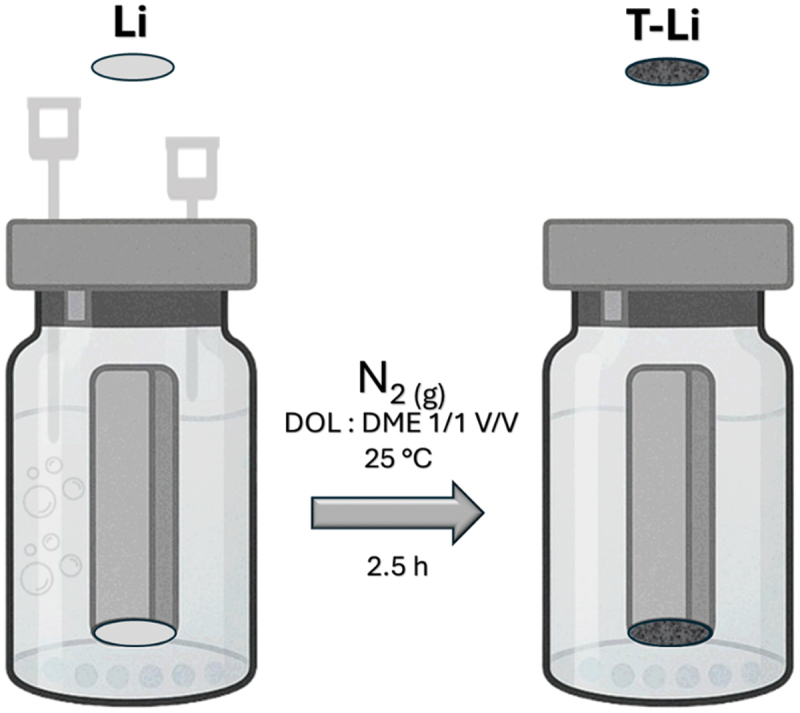
Processing scheme for the preparation of the T-Li sample.

A lithium chip (Cambridge Energy Solutions Cambridge, United Kingdom, 0.45 mm thick) was cut inside an argon-filled glovebox and was attached on top of a stainless steel (SS) piston. The piston was put inside a glass vial containing an equivolume solution consisting of 1,2-dimethoxyethane (DME, ≥99%, Sigma-Aldrich, Germany) and DOL (≥99,8%, Sigma-Aldrich) and sealed inside an Ar-filled glovebox (O_2_ < 0.1 ppm and H_2_O < 0.1 ppm, Mbraun Labmaster SP). In order to prevent the lithium from stacking at the bottom of the vial, glass balls were used to physically separate the lithium from the glass flask. The solution was later bubbled with nitrogen gas for 2.5 h (Figure 1(a)). The shiny surface of lithium gradually became dark grey, indicating that the protective layer is being grown. The vial with the N_2_-treated Li sample was then bubbled with Ar for 15 min to remove N_2_ present in the vial atmosphere and transferred inside the glovebox. DME was included in the pretreatment solvent to maintain the same DOL:DME (1:1 v/v) ratio as in the electrolyte, ensuring consistency between the pretreatment medium and the electrochemical environment. DME itself is chemically inert towards both lithium and nitrogen under the employed conditions and does not participate in the surface reaction. Its presence moderates the reactivity of DOL by lowering solvent polarity and viscosity, thereby preventing the formation of excessively resistive coatings. The preparation procedure was also tested without nitrogen bubbling and with argon bubbling, which is completely inert towards lithium. Only the sample prepared with nitrogen bubbling exhibited the formation of a dark grey layer, as observed visually. As shown in Figure S1, all the other samples remained shiny. These observations suggest that the formation of the layer depends on the presence of N_2_.

### 2.2. S/C composite cathode preparation

The recipe for the fabrication of the cathode was adopted from elsewhere and slightly modified in terms of the sulphur content [34]. A slurry containing 80% S/C, 10% conductive additive (KS6, TIMCAL, Imerys Graphite & Carbon, Switzerland) and 10% binder (PVdF-HSV 900, Kynar, Arkema Inc. Pennsylvania) was prepared and spread on a sheet of Al, which was previously etched with KOH, using a Hohnsen (Japan) MC20 minicoater. The velocity was 0.3 cm s^−1^ and the bar gap was adjusted to 500 μm (20 mil). After drying for about 1 h at room temperature, the electrode coatings were left to dry at 70°C. The electrodes were then cut to the desired size, pressed under 1 t for 1 min and dried under vacuum in a Buchi oven at 50°C for 12 h. The S/C composite cathode with a sulphur mass content of 59% and active mass loading of 1.74 mg cm^−2^ was obtained. The lower bound of the operational voltage was 1.9 V.

### 2.3. Chemical–physical characterization

Before performing any chemical–physical characterization, the T-Li was dried in the antichamber of the glovebox under vacuum for 30 min. Scanning electron microscopy (SEM) images were obtained using a ZEISS Crossbeam 550 equipped with a Gemini II field emission (FE-SEM) column (Carl Zeiss Meditec AG, Germany). Energy dispersive spectra (EDS) were acquired using a Bruker X-Flash 7100 Energy Dispersive Spectrometer. FTIR-ATR was performed with a spectrometer FT-IR ATR Agilent Cary 630 (Agilent Technologies, California) in the range 600 cm^−1^/4000 cm^−1^. XRD measurements were carried out with the Empyrean diffractometer (Malvern Panalytical Ltd., Malvern, United Kingdom), with a Cu-*K*α X-ray tube (Cu LFF-HR), a diffracted beam optic, and a 3D detector. The 2*θ* interval was 30°/70° using an XYZ sample stage. The Li and T-Li samples for postmortem analyses were prepared onto a thin, transferable stainless steel current collector instead onto stainless steel pistons to avoid further manipulations of the samples. After treatment and/or cycling, the sample was transferred to the SEM holder without detachment from the current collector, preventing any mechanical deformation of the soft lithium surface. This approach eliminated the bending-induced cracks observed in preliminary samples and allowed direct observation of the as-prepared T-Li coating.

XPS analyses were carried out with an EnviroESCA system (SPECS Surface Nano Analysis GmbH, Germany), featuring an Al-*K*α radiation source (photon energy: 1486.6 eV). Measurements were conducted under vacuum at a pressure of about 10^−6^ mbar. High-resolution spectra were collected using a pass energy of 30 eV, with an integration time of 0.2 s per step and an energy step size of 0.1 eV. To correct for charging effects, the binding energy (BE) scale was calibrated using the C 1s peak of adventitious carbon, set at 284.8 eV [35]. Data processing and spectral fitting were performed with Keystone software (Specs), employing a Shirley background subtraction and Voigt function line shapes [36]. Elemental quantification was based on parameters supplied by the instrument manufacturer.

### 2.4. Electrochemical measurements

The interfacial stability of pristine Li and T-Li was studied through symmetric PTFE cells (Bohlender GmbH, Germany), with Whatman GF/A separator and 400 μL of the electrolyte containing 1 M LiTFSI in DOL/DME (1:1 by volume) with LiNO_3_ additive at 0.45 M concentration. Lithium (10 mm) symmetric cells were characterized with a BioLogic VSP potentiostat/galvanostat (Biologic, France) using the following test protocol: 25 deposition/stripping cycles at 0.125 mA cm^−2^; 25 deposition/stripping cycles at 0.250 mA cm^−2^; 25 deposition/stripping cycles at 0.500 mA cm^−2^ and 425 deposition/stripping cycles at 0.125 mA cm^−2^; each cycle is composed of 30-min deposition and 30-min stripping. EIS measurements were carried out over a frequency range from 100 kHz to 100 mHz, using a 10-mV perturbation amplitude around the open-circuit voltage (OCV), and collecting 20 data points per frequency decade. The resulting spectra were analysed using equivalent circuit models composed of resistive (*R*) elements and constant-phase elements (*Q*). The use of *Q*, rather than an ideal capacitor, accounts for non-ideal capacitive behaviour typically arising from surface roughness or heterogeneity. The impedance of the constant-phase element is described by the expression: *Z*(CPE) = 1/[*Q* × (*iω*)^a^], where *Q* is expressed in units of F s^1–*a*^ (or Ω^−1^ s^*a*^), and *ω* is the angular frequency (*ω* = 2*πν*). From these fits, the effective capacitance can be estimated using the relation *C* = (*Q* × *R*^1–*a*^)^1/*a*^ [37,38]. Each RC pair defines a time constant *τ* = RC, which corresponds to a characteristic frequency *ν* = 1/(2*πτ*) observable in the impedance spectrum. The experimental protocol included the following steps: EIS (cycle number 0); 10 GCPL cycles at 0.125 mA cm^−2^; EIS (cycle number 10); 15 GCPL cycles at 0.125 mA cm^−2^; EIS (cycle number 25); 25 GCPL cycles at 0.250 mA cm^−2^; EIS (cycle number 50); 25 GCPL cycles at 0.125 mA cm^−2^; EIS (cycle number 75); 50 GCPL cycles at 0.125 mA cm^−2^; EIS (cycle number 125); prolonged GCPL cycling at 0.125 mA cm^−2^ with EIS recorded every 100 cycles.

Li//S cells were assembled in El-Cell ECC.Std cells (EL-Cell GmbH, Hamburg, Germany) with lithium (modified or pristine) as anode, S/C composite as cathode and a Whatman GF/A separator. About 1 M LiTFSI 0.45 M LiNO_3_ in DOL/DME (1:1 by volume) was used as the electrolyte. Active material stability tests of Li//S cells were monitored via galvanostatic charge/discharge at C/10. Rate capability tests were conducted using the following protocol: 1 discharge half-cycle at C/20, 4 cycles at C/10, 4 cycles at C/5, 4 cycles at C/3, 4 cycles at C/5, 12 cycles at C/10, and 10 cycles at C/5.

## 3. Results and discussion

### 3.1. Chemical–physical characterization

**Figure 2.**
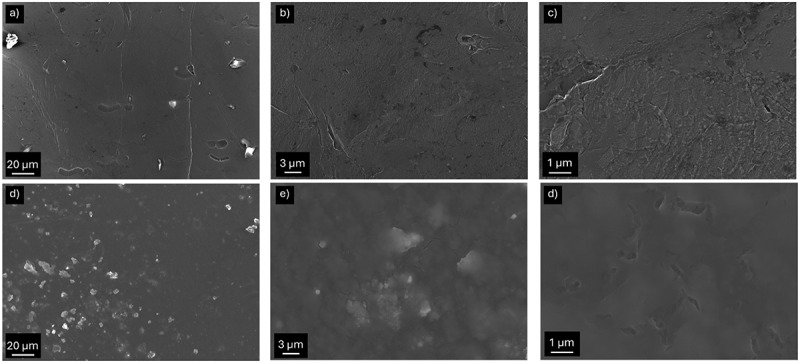
SEM images at different magnifications of pristine lithium electrode (a–c) and after the treatment (T-Li sample) (d–f).

The morphology of pristine and T-Li was investigated by SEM, as shown in Figure 2. Panels a–c show the surface of pristine lithium metal at increasing magnifications. The electrode appears relatively smooth and compact, with minor surface defects, such as scratches and superficial cracks, typical of fresh lithium handled under inert conditions. Figure 2(d–f) display the morphology of the T-Li surface. The treatment results in the formation of a homogeneous and continuous coating that uniformly covers the lithium substrate. The surface consists of densely packed, fine grains, with no evidence of cracks or uncovered areas. EDS spectra performed on the sample show that large microparticles have a large carbon and oxygen content (Figure S2). This tridimensional structure could be beneficial for the deposition-stripping process thanks to the increase in surface area, thereby mitigating the formation of dendrites and promoting more stable electrode behaviour. Additionally, the carbonaceous structure is expected to act as an artificial SEI, limiting the electrolyte consumption during electrochemical tests.

**Figure 3.**
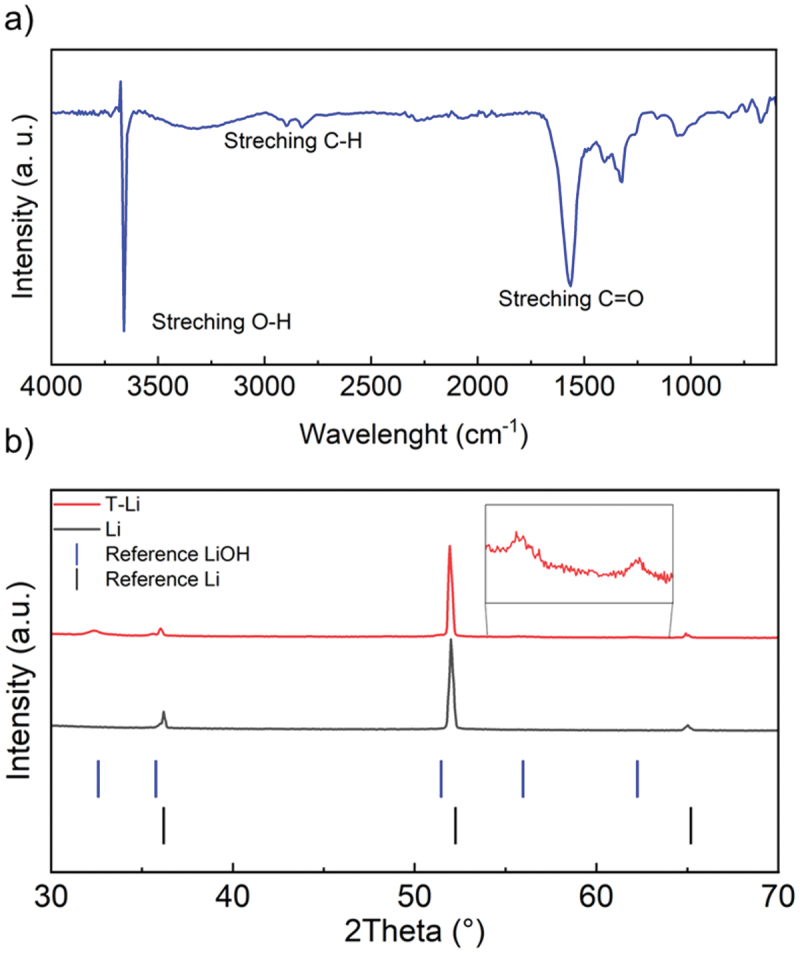
(a) FTIR-ATR spectrum of the coating scraped from the T-Li electrode; (b) XRD patterns of T-Li, Li, and reference sticks of Li and LiOH (Only peaks with relative intensity higher than 0.5% have been considered).

The coating on the T-Li surface was scratched and analysed using FTIR-ATR spectroscopy, as shown in Figure 3(a), to identify functional groups that could provide further insight into the nature of the treated surface. A broad and intense absorption band appears around 3400 cm^−1^, likely corresponding to hydroxyl groups, with the hydrogen atoms not strongly involved in hydrogen bonding. Peaks observed in the 2950–2850 cm^−1^ region are characteristic of C–H stretching vibrations, indicating the presence of alkyl chains. A sharp and intense peak at 1710 cm^−1^ is attributed to C=O stretching, suggesting the presence of carbonyl-containing species. Additionally, several medium-intensity bands in the 1600–1500 cm^−1^ region correspond to C=C stretching vibrations, indicative of alkenes. The simultaneous presence of O–H and C=O groups points towards carboxylic acids; however, the formation of inorganic species such as LiOH and Li_2_CO_3_ cannot be excluded, as also reported by Fiedler and co-workers [39].

The presence of LiOH was confirmed by XRD analysis (Figure 3(b)). Diffractograms of pristine lithium and T-Li were recorded to assess changes in the crystalline structure following the surface treatment. The XRD patterns exhibit clear differences: the diffraction peaks of pristine lithium match well with the standard reference pattern for metallic Li (00-001-1131, Cubic, *Im*-3*m*), while the T-Li sample displays additional peaks that can be attributed to LiOH (01-085-0777, Tetragonal, *P*4*nmm*). Although these additional peaks are of relatively low intensity, the most characteristic reflections of LiOH are clearly visible. This observation is consistent with the broad O–H stretching band identified in the FTIR-ATR spectrum, further supporting the presence of hydroxyl-containing species on the T-Li surface.

XPS studies reveal that the surface of the investigated lithium-based materials is predominantly composed of carbon, oxygen, and lithium, as expected (see survey spectra in Figure S3 and Table S1). Traces of F, Cl, Si, Zn, and Ca are also detected, likely due to residual impurities present on the surface of the starting materials. Nitrogen and sulphur are observed only in the cycled samples (i.e. Li_cyc_ and T-Li_cyc_). Both nitrogen- and sulphur-containing species are attributed to decomposition products of the TFSI^−^ anion in the electrolyte, forming part of the solid electrolyte interphase (SEI).

The surface compositions of both Li and T-Li samples are similar, with approximately 15 at% C, 41 at% O, and 41 at% Li, indicating that the N_2_ bubbling treatment alone does not significantly alter the total elemental surface composition of lithium metal. In contrast, electrochemical cycling results in a carbon enrichment and a lithium depletion, suggesting progressive SEI formation on the lithium surface. This effect is more pronounced in T-Li_cyc_, which shows a strong accumulation of carbonaceous species, likely due to electrolyte degradation.

**Figure 4.**
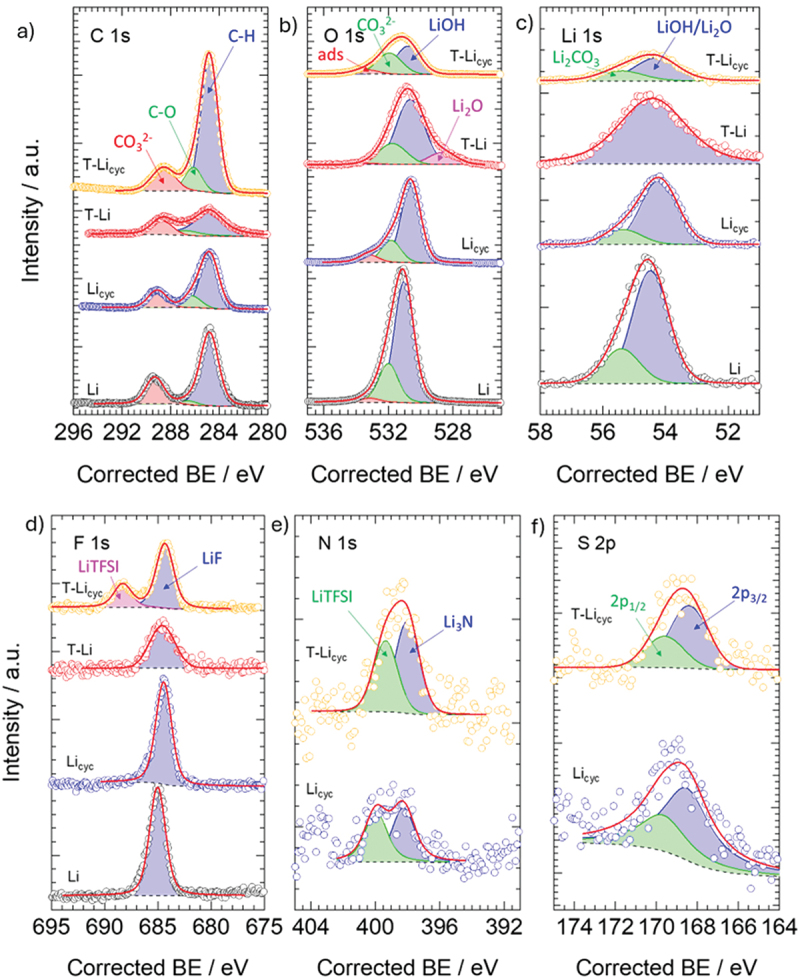
High resolution XPS analysis and fitting of (a) C 1 s, (b) O 1s, (c) Li 1s, (d) F 1 s, (e) N 1s, and (f) S 2p spectral regions. Assignment is highlighted in each panel. Markers indicate the experimental curves, and the red line is the fitting result.

High-resolution C 1s spectra reveal three main carbon species (see Figure 4(a)): (i) C–H (sp_3_-hybridized hydrocarbons, ca. 284.8 eV), (ii) C–O or C–F groups (ca. 286.5 eV), and (iii) carbonates and oxidized carbons (including Li_2_CO_3_, ca. 288.3 eV) [40–43]. C–H species are the most abundant (>50 at%), followed by carbonates and oxidized carbons, while C–O/C–F species are the less prevalent (see Table S2). In the O 1s region (see Figure 4(b)), three main components are assigned to LiOH (ca. 530.8 eV), carbonates (ca. 531.9 eV), and adsorbed oxygen (ca. 533.2 eV) [40–42]. Notably, the T-Li sample displays an additional feature at 528.6 eV, attributed to Li_2_O – absent in the other samples. Oxygen species follow the relative trend: ads < Li_2_O < CO_3_^2−^ < LiOH (see Table S3). The Li 1s spectra (see Figure 4(c)) exhibit two primary features: (i) LiOH/Li_2_O (ca. 54.3 eV) and (ii) Li_2_CO_3_ (ca. 55.4 eV) [40–42]. In the F 1s region (see Figure 4(d)), all samples exhibit a peak at ca. 684.6 eV, assigned to LiF. LiF likely originates from decomposition of the LiTFSI electrolyte or contamination from the pristine metal. The T-Li_cyc_ sample uniquely shows a second peak at 688.3 eV, corresponding to TFSI-derived degradation products [40]. The N 1s spectra (see Figure 4(e)) reveal two peaks in the cycled samples, Li_3_N (ca. 398.1 eV) and LiTFSI residues (ca. 399.7 eV) [40], which confirms SEI formation from the electrolyte. These features are absent in non-cycled samples. Similarly, the S 2p region (see Figure 4(f)) displays spin – orbit-split peaks assigned to TFSI-derived sulphur species, found in cycled samples.

XPS analysis reveals that electrochemical cycling profoundly alters the surface composition of lithium metal anodes by promoting the formation of a complex SEI enriched in carbonaceous and inorganic species. In particular, cycled samples (Li_cyc_ and T-Li_cyc_) exhibit a substantial increase in surface carbon (up to ~46 at%), coupled with a marked depletion of lithium and oxygen. This suggests progressive accumulation of electrolyte decomposition products, such as lithium alkyl carbonyls and carbonates, polyethers, and residual solvent derivatives, on the lithium surface. The presence of nitrogen and sulphur species, attributed to the reduction and fragmentation of the TFSI^−^ anion, further supports the formation of an ionically and chemically heterogeneous SEI. Notably, the T-Li_cyc_ sample displays characteristic XPS signals of both Li_3_N and LiTFSI, indicating simultaneous reductive decomposition and incorporation of nitrogen-containing fragments within the interphase. The T-Li sample maintains a surface composition comparable to pristine Li in terms of total C, O, and Li content. However, high-resolution XPS reveals a pronounced Li_2_O component (528.6 eV), the absence of a well-defined Li_2_CO_3_ peak in the Li 1s spectral region, and broader signals overall – features consistent with the formation of a thick, electronically insulating SEI layer.

Altogether, these results highlight how electrochemical cycling and gas environment during surface engineering influence the surface chemistry of lithium metal surface. The proposed treatment emerges as a valuable strategy to modulate SEI composition, stabilize the interface, and potentially enhance lithium metal anode performance.

The results shown in XRD, FTIR, and XPS suggest a complex interplay between lithium metal, nitrogen gas, and DOL, likely leading to both ring-opening polymerization and the formation of nitrogen-containing species. While the direct formation of crystalline Li_3_N from Li and N_2_ typically requires elevated temperatures [44], several studies have demonstrated that transient Li–N intermediates and amorphous nitrides can form under electrochemical or low-temperature conditions (Li-mediated nitrogen reduction) [45,46]. These species could serve as reactive bases or nucleophiles in the presence of ether solvents such as DOL. The pathway can proceed through ionic mechanisms, with lithium alkoxides or lithium amides as initiators [47,48]. In the current system, lithium alkoxides or lithium amide/imide intermediates generated in the presence of N_2_ could similarly initiate DOL polymerization, yielding poly-DOL-type surface films.

**Figure 5.**
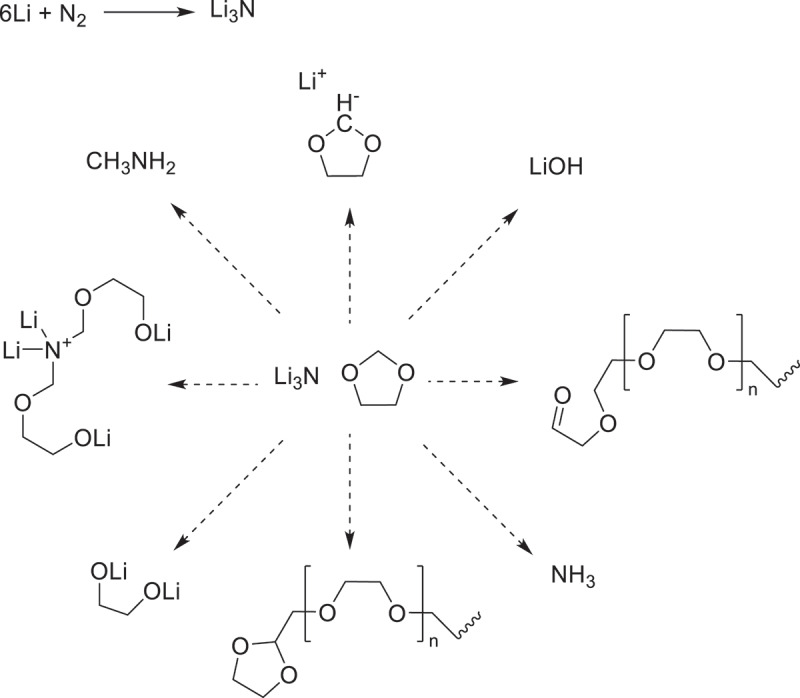
Possible reaction products of lithium, nitrogen, and 1,3-dioxolane.

However, it is also plausible that competitive radical pathways may be involved. Radical-mediated decomposition of DOL under reductive conditions has been proposed in lithium metal batteries, potentially accelerated by nitrogen-derived radicals or solvated electrons [49]. Such radical pathways could promote both the breakdown of DOL and the evolution of volatile nitrogen-containing species, such as ammonia or methylamine, consistent with our observation that nitrogenous compounds are absent before cycling. Moreover, prior studies on LiNO_3_-containing Li//S electrolytes have shown that nitrogen-rich interphases (Li_3_N, LiN_*x*_O_*y*_, or amide species) stabilize Li metal and suppress polysulfide shuttling [50]. This [51] supports [52] our interpretation that transient Li–N species formed in the DOL/N_2_ environment could yield analogous products, contributing to interphase stabilization and parasitic reaction mitigation. The ^1^H NMR spectrum of the powder collected from the T-Li surface, recorded in DMSO-d_6_ (Figure S4), reveals the presence of soluble organic species derived from DOL degradation. The signals indicate ring opening and oxidative cleavage of the dioxolane, leading to the formation of aldehydes, glycols, and ether derivatives, consistent with polymerization and oxidation processes occurring at the lithium surface. Since multiple reaction pathways are possible, Figure 5 shows the reaction products that are compatible with our observations.

**Figure 6.**
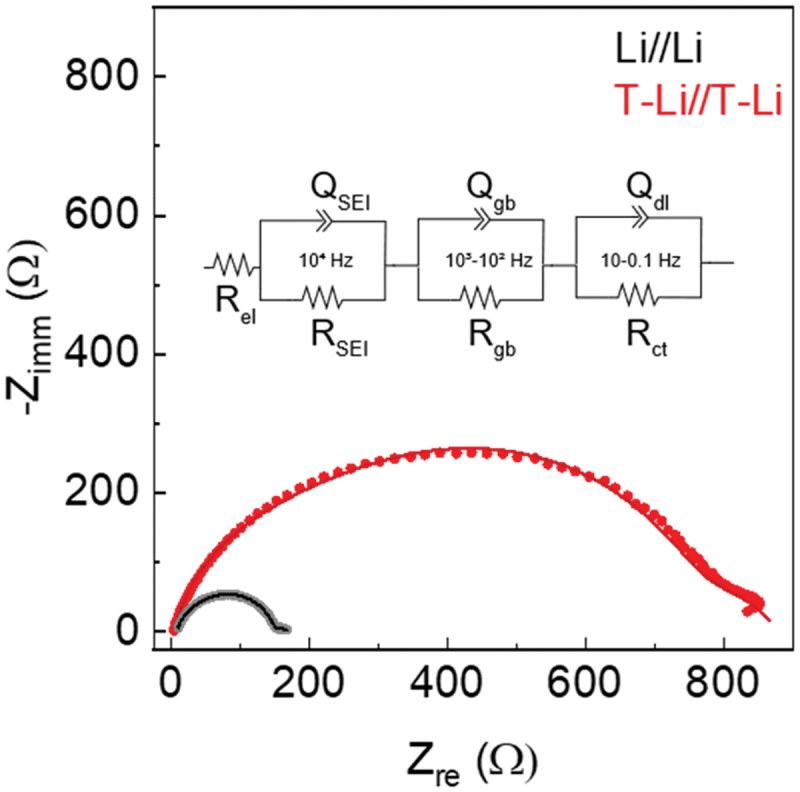
Impedance spectra and fitted curves for Li//Li and T-Li//T-Li symmetric cells at fresh state. The solid lines are the data fittings.

The characteristics of the interphase after the treatment were assessed by collecting impedance spectra immediately after cell assembly (Figure 6). The impedance data were analysed using the equivalent circuit: *R*_el_(*R*_SEI_*Q*_SEI_)(*R*_gb_*Q*_gb_)(*R*_ct_*Q*_dl_), with fitting details reported in Table 1. In this model, *R*_el_ represents the bulk resistance of the electrolyte; *R*_SEI_ and *Q*_SEI_ correspond to the impedance contributions from the solid electrolyte interphase (SEI); *R*_gb_ and *Q*_gb_ are attributed to impedance at grain boundaries within the SEI or at the SEI–lithium interface, while *R*_ct_ and *Q*_dl_ describe the charge transfer resistance and the associated double-layer capacitance at the electrode–electrolyte interface [37,38]. These components were assigned based on their characteristic frequency domains in the impedance spectra. Notably, the semicircle associated with the SEI can be easily distinguished due to its relatively low capacitance compared to the electrical double layer at the lithium interface (10^−6^ F vs. 10^−2^ F).

**Table 1.** Results from the fitting of the EIS reported in Figure 6.

	R_el_ Ω	R_SEI_ Ω	Q_SEI_ 10^−6^ F s^a-1^	a_SEI_	R_gb_ Ω	Q_gb_ 10^−6^ F s^a-1^	a_gb_	R_ct_ Ω	Q_dl_ 10^−3^ F s^a-1^	a_dl_
Li//Li	9.22	127.7	2.109	0.865	11.6	1.21	0.999	22.8	13.7	0.478
	±0.06	±0.2	±0.004	±0.001	±0.1	±0.02	±0.003	±0.2	±0.1	±0.001
T-Li//T-Li	4.14	554.96	9.2	0.859	200	4.65	0.870	117	1.8	0.696
	±0.13	±2.49	±0.1	±0.001	±3	±0.09	±0.002	±5	±0.2	±0.002

The fitting results are summarized in Table 1 and show that the cell with treated lithium electrodes (T-Li//T-Li cell) shows significantly lower bulk resistance, likely due to improved contact, better electrolyte penetration, possibly facilitated by the artificial layer. *R*_SEI_ and *Q*_SEI_ suggest that the artificial layer leads to a thicker or denser SEI, potentially offering more stability or protection, but at the cost of higher initial impedance. Additionally, a higher *Q*_SEI_ could imply an increase in the surface area or a more porous interphase, depending on the morphology that can be originated by the reaction of lithium during the treatment. *R*_gb_ in T-Li//T-Li cell was approximately 17 times higher than in bare Li, suggesting that the artificial layer introduces more complex or resistive grain boundary interfaces, possibly due to the polycrystalline or layered morphology of the coating. This interpretation is supported by morphological and structural characterizations. SEM images (Figure 2(d–f)) reveal a surface composed of fine interconnected grains and domains, indicative of the presence of grain boundaries across the coating. Such features are consistent with the XRD pattern of T-Li (Figure 3(b)), which displays distinct reflections assigned to crystalline LiOH, confirming the presence of ordered domains within the interphase. Moreover, XPS spectra (Figure 4(b–c)) corroborate the coexistence of LiOH and Li_2_O phases, both typically forming polycrystalline aggregates. The increase of the *R*_ct_ in the T-Li//T-Li cell indicates that charge transfer across the interphase is hindered in T-Li, possibly due to an initial passivation effect. This could be beneficial in long-term cycling by providing better stability, but it results in higher initial impedance and lower reactivity. In contrast, the bare lithium cell shows lower resistances but likely suffers from less controlled SEI growth and potentially lower long-term stability.

### 3.2. Symmetric cell performance

**Figure 7.**
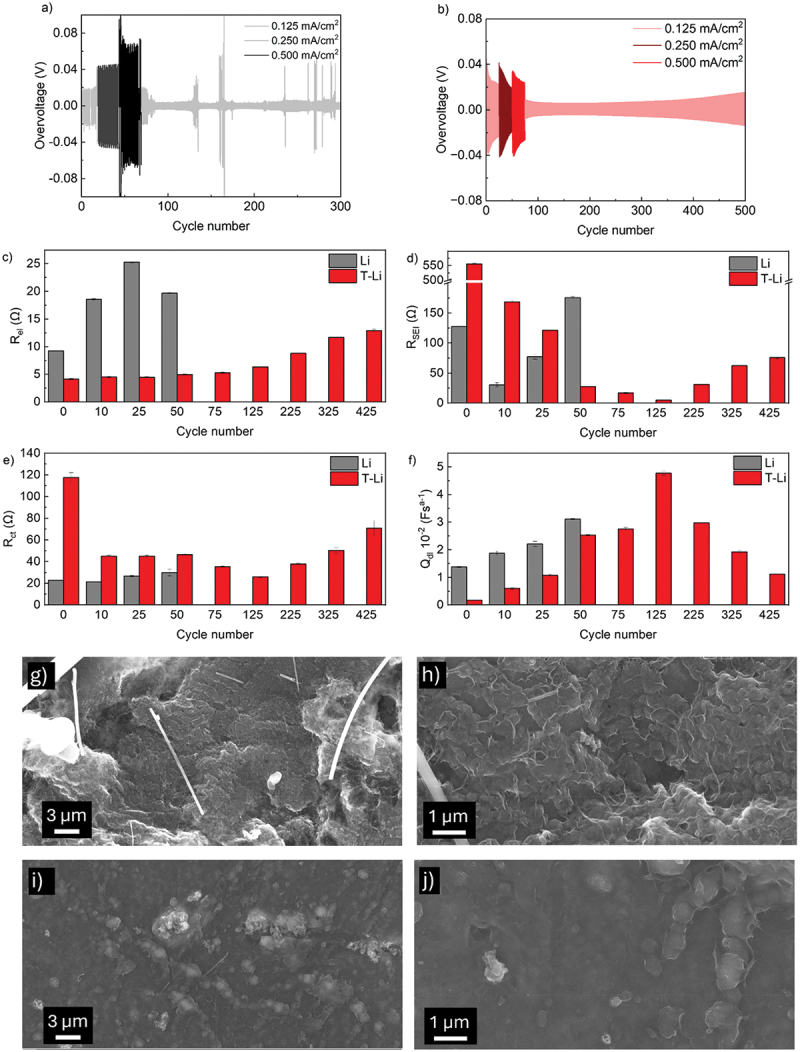
Voltage profiles in 1 M LiTFSI DOL:DME with LiNO_3_ 0.45 M of Li//Li symmetric cells at different current density (0.5 h stripping/0.5 h plating): (a) Li//Li; (b) T-Li//T-Li; (c–f) evolution of *R*_el_, *R*_SEI_, *R*_ct_, and *Q*_dl_ over cycling for symmetric Li//Li and T-Li//T-Li cells; (g–j) SEM images of Li (g, h) and T-Li (i, j) electrodes after 20 deposition/stripping cycles at 0.125 mA cm^−2^.

Initially, the impact of the modification has been investigated in Li symmetrical cells. Figure 7 summarizes the overvoltage profiles of symmetric cells during 500 cycles. As is evident, the symmetric cell with T-Li exhibited smoother and more stable overvoltage profiles, indicating a stabilized interface (Figure 7(b)). It should be noted that pristine lithium cells, without the coating and using only 0.45 M LiNO_3_ instead of the commonly used 0.8 M, did not display stable or reproducible behaviour, with several short-circuit events occurring (Figure 7(a)). Among the various repetitions, all cells showed this unstable trend, except for the cell reported in Figure S5, which cycled for 500 cycles but exhibited an exceptionally high overvoltage.

Li//Li cell shows a stable profile until 150 cycles. Thereafter, the overvoltage increases over the next 100 cycles. This behaviour could be due to unstable SEI formation that triggers lithium exposure to the electrolyte and consequently further electrolyte consumption. This process happens in each cycle and consumes the electrolyte. Besides, the isolation of lithium dendrites wrapped by the SEI layer forms dead lithium that piles up on the electrode surface. These two factors play a significant role in the voltage build-up. On the other hand, T-Li//T-Li cell shows a stable overvoltage profile even at different current densities. From the 250th cycle, overvoltage progressively increases probably due to the accumulation of dead lithium.

To delve deeper into the electrochemical functioning of lithium/electrolyte interface with and without the protective layer, EIS tests were carried out before cycling and after several cycles (Figure S6). As depicted in Figure 6, EIS were fit considering *R*_el_(*R*_SEI_*Q*_SEI_)(*R*_gb_*Q*_gb_)(*R*_ct_*Q*_dl_) equivalent circuit and all the details of the fitting are reported in Tables S5 and S6. Data for the Li//Li cells were limited to the first 50 cycles because, as previously reported, the system is not fully stable when the current is increased to 0.5 mA cm^−2^. In Figures 7(c–f) and S6, the evolution of the different equivalent circuit components has been reported. Li//Li cell shows an increase in *R*_el_ during the first 50 cycles (Figure 7(c)) indicating electrolyte consumption due to the SEI formation. In contrast, T-Li//T-Li cell maintains a lower and more stable *R*_el_ throughout cycling, pointing to a reduced electrolyte decomposition, which can be ascribed to the presence of the artificial protective layer. After 125 cycles, the *R*_el_ progressive increase can be due to the accumulation of dead lithium as suggested by the overvoltage profile reported in Figure 7(b).

In the T-Li//T-Li cell *R*_SEI_ (Figure 7(d)) results initially exhibit a higher *R*_SEI_ due to the pre-formed interlayer but rapidly stabilize after ca. 50 cycles. On the other hand, *R*_SEI_ of the Li//Li cell gradually increases, reflecting continuous SEI growth from ongoing electrolyte decomposition.

Figure 7(e) displays the evolution of the *R*_ct_. When lithium is treated, the initial *R*_ct_ results are higher probably because of the blocking nature of the artificial layer, which hinders the lithium-ion transport. Among cycling, *R*_ct_ drops and stabilizes, suggesting early interface adaptation. In contrast, bare Li cell shows an overall lower *R*_ct_ in the interval considered.

Li//Li shows increasing *Q*_dl_ consistent with surface roughening and increasing surface area matching with a dendritic growth (Figure 7(f)). Also, it must be noted that Li//Li cells showed an overall instability after the cycles at 0.500 mA cm^−2^. Analogously, the T-Li containing cell exhibits a progressive increase in *Q*_dl_ during cycling at increasing current, indicating surface roughening. However, *Q*_dl_ decreases after cycle 125, when the current is lowered to 0.125 mA cm^−2^. The lower *Q*_dl_ values suggest that, during the prolonged cycles at 0.125 mA cm^−2^, the lithium surface becomes more uniform and smoother.

Evolution of *Q*_SEI_, *R*_gb_, and *Q*_gb_ over cycling for symmetric Li//Li and T-Li//T-Li cells are reported in Figure S7. As already mentioned, the comparison of *R*_gb_ values reported in Table 1 suggests the presence of a grained SEI on T-Li. It must be noted that this resistance decreases over cycling pointing a readjustment of the SEI morphology. On the other hand, the same parameter in the Li//Li cell increases because of the high reactivity of the bare metal, which leads to an uncontrolled SEI formation in the early stage of the cycling. The *Q*_dl_ and *Q*_SEI_ show stable behaviour. Overall, while T-Li presents initially higher resistances, it rapidly stabilizes and enables more controlled SEI formation and improved properties compared to bare Li, which suffers from progressive degradation.

To evaluate the effect of the surface treatment on the electrochemical stability and lithium deposition behaviour, post-mortem SEM analyses were carried out on Li electrodes after 20 plating/stripping cycles at 0.125 mA cm^−2^ in Li/Li symmetric cells. Figure 7(g,h) shows the surface morphology of pristine Li electrodes. The surface appears highly irregular and porous, with extended regions of rough and fractured deposits, suggesting non-uniform lithium plating and localized stripping. In contrast, Figure 7(i,j) display the surface of the T-Li electrode cycled under identical conditions. Here, the morphology is significantly smoother and more compact, with a continuous granular texture that resembles pristine samples with no visible dendritic protrusions. The coating remains largely intact, suggesting that the treated surface promotes more homogeneous Li deposition and stripping.

### 3.3. Application of T-Li in Li//S batteries

As a proof of concept, the feasibility of the protected lithium in a full cell was investigated. The T-Li was used as the anode for Li//S cell at room temperature.

**Figure 8.**
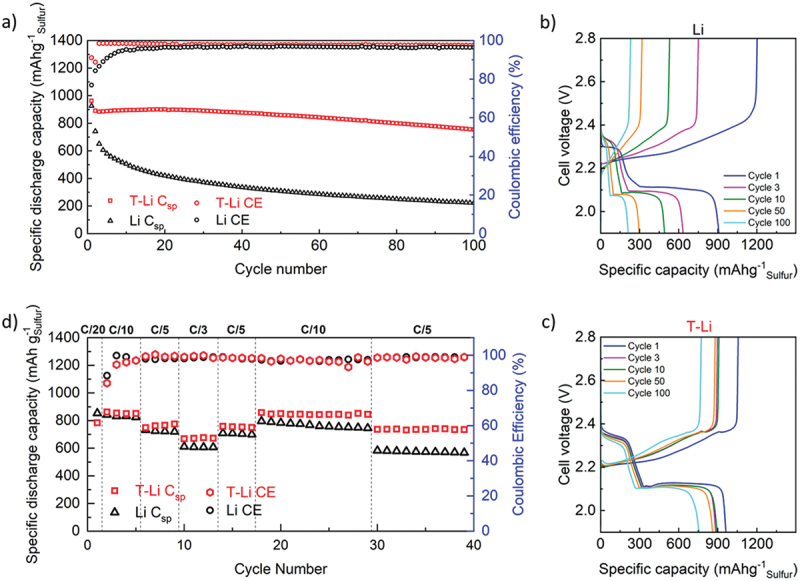
Stability test: (a) discharge specific capacity over cycles of T-Li//S and Li//S cells at C/10; (b–c) charge/discharge profiles of Li//S and T-Li//S cells at selected cycles; and (d) rate performance (1*C* = 1672 mA g^−1^) of Li//S (Black) and *T*- Li//S (red). .

Figure 8(a) depicts the cycling performance of Li//S full cells with treated lithium and pristine lithium anodes at C/10 (1C = 1672 mA g^−1^). At first glance, it is evident that T-Li//S cells show a higher discharge capacity with superior cycling stability compared to the Li//S cell over 100 cycles. The cell with the T-Li layer showed an average coulombic efficiency of 98% compared to 95.4% for the cell with pristine lithium. Figure 8(b,c) compares the charge/discharge voltage profiles of the Li//S cells as a function of specific capacity at selected cycles. T-Li//S cell delivered an initial discharge capacity of 962 mAh g^−1^ with the coulombic efficiency of 91% compared to corresponding values of 916 mAh g^−1^ and 77% for pristine Li//S cell. Representative voltage profiles of Li//S cells with bare Li and T-Li are reported in Figure 8(b,c), respectively. The ≈200 mAh g^−1^ overcharge observed with bare Li in the first cycle (Figure 8(b)) indicates the occurrence of parasitic reactions. Typically, initial polysulfide shuttling, enabled by the poorly developed SEI, together with uncontrolled SEI growth and electrolyte decomposition at the anode have been reported as the main causes of the first cycle overcharge [50,53]. Such processes draw excess anodic charge without effective sulphur utilization, consistent with previous observations that LiNO_3_ concentrations lower than 0.8 M cannot fully suppress first-cycle parasitic reactions [50,53]. In contrast, since SEI has already been formed in T-Li, its interphase efficiently limits polysulfide reduction, reduce the shuttling (visible as a distinct high-voltage oxidation plateau at ~2.35–2.40 V), and yields a higher first-cycle CE (91% vs. 77%). At the third cycle, the T-Li//S cell shows rapid activation with a stable capacity of around 850–900 mAh g^−1^ and a coulombic efficiency close to 98–99%. The voltage–capacity profiles retain the two typical discharge plateaus, reflecting the reversible oxidation of long-chain polysulfides and limited polarization. In contrast, the cell with unmodified lithium already exhibits a marked decline in capacity to about 700–800 mAh g^−1^, a coulombic efficiency below 95%, a broader hysteresis, and a shortened high-voltage plateau, which are all indicative of polysulfide shuttling and an unstable SEI.

Among cycling, this behaviour remains consistent as the specific capacity of the cell with bare Li further drops, while the T-Li//S cell maintains capacity around 900 mAh g^−1^ with minimal voltage shifts and nearly 99% coulombic efficiency. At 50th, T-Li//S delivered 861 mAh g^−1^ (90% capacity retention), while the cell with unmodified lithium showed a specific discharge capacity of 305 mAh g^−1^ (33% capacity retention). Finally, after 100 cycles, the cell with T-Li provides a discharge capacity of 752 mAh g^−1^ with the capacity retention of 78%. The presence of high-voltage plateaus (between 2.35 and 2.4 V) at the end of the charging step is noticed for the T-Li//S cell that can be assigned to the reversible oxidation of the long-chain polysulfides. Taken together, the fresh-state EIS and the superior long-term electrochemical performance indicate that the treatment forms a more robust passivation layer that reduces the direct reduction of soluble polysulfides at the anode. During cycling, the rapid stabilization of interfacial resistances, the preservation of *R*_el_, and the persistence of the high-voltage polysulfide oxidation plateau (~2.35–2.40 V) collectively point to suppressed polysulfide shuttling and diminished electrolyte consumption relative to bare Li.

Figure 8(d) presents the rate capability experiments of Li//S (Black) and T-Li//S (red) cells. An initial discharge capacity of 853 mAh g^−1^ was obtained for the first half-cycle discharge of the Li//S cell at C/20. Then, four cycles at C/10 delivered an average discharge capacity of 833 mAh g^−1^ which dropped to 725 mAh g^−1^ at C/5 and later to 609 mAh g^−1^ at C/3. By moving back to C/5 for four cycles and C/10 for 12 cycles, average discharge capacities of 705 mAh g^−1^ and 787 mAh g^−1^ were obtained, respectively. The latter capacities are lower than the former corresponding values, which suggest Li//S cell could not successfully retrieve the initial values. Moreover, upon cycling at C/10, a descending trend was observed in discharge capacities that was also repeated through shifting to C/5. On the other side, for the T-Li//S cell, the lower discharge capacity at C/20 can be attributed to the lower initial OCV value of 2.32 V. An average discharge capacity of 839 mAh g^−1^ related to four charge/discharge cycles at C/10 is slightly higher than the value previously observed for Li//S cell. Upon shifting to higher rates of C/5 and C/3, average discharge capacities of 749 mAh g^−1^ and 660 mAh g^−1^ were obtained, respectively, which are both higher than their counterparts for the Li//S cell. Eventually, by decreasing the rates to C/5 and C/10, the average capacities of 740 mAh g^−1^ and 835 mAh g^−1^ were successfully recovered. The final increase in the *C* rates to C/5 for an additional 10 cycles clearly demonstrates the robustness and electrochemical stability of the T-Li. Indeed, T-Li//S delivered 723 mAh g^−1^, a value very similar to the previous two measurements at this C-rate. On the other hand, the value of 572 mAh g^−1^ was obtained for the Li//S cell indicating a dramatic drop in its performance. Considering that the only difference between the two experiments refers to the anode, the superior rate capability performance of T-Li//S cell can only be ascribed to the use of T-Li in which the treatment stabilizes Li surface and enhances cycling performance.

**Figure 9.**
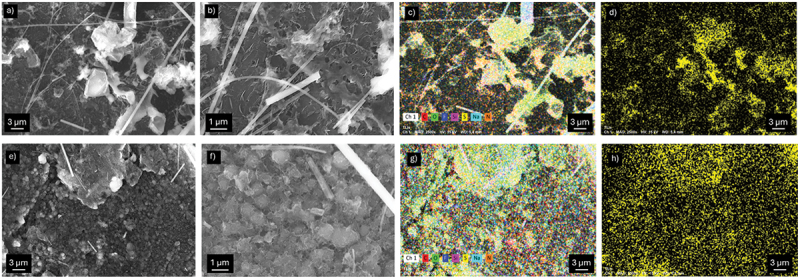
Post-mortem SEM and EDX analyses of pristine Li (a–d) and T-Li (e–h) electrodes after 20 cycles in Li//S cells at 1C.

To further investigate the effect of the surface modification on the interfacial stability in Li//S cells, post-mortem SEM and EDX analyses were performed on pristine Li and T-Li electrodes after 20 cycles at 1*C* (Figure 9). Figure 9(a–d) shows the morphology and elemental distribution of the pristine Li electrode, which appears covered by reaction products, in agreement with the features previously observed in Li symmetric cells. The EDX maps confirm the presence of sulphur with an inhomogeneous distribution and a total sulphur content ranging between 1.7 and 2.6 at% across different regions. This non-uniform accumulation indicates the formation of sulphur-rich deposits derived from the polysulfide shuttling, leading to the progressive accumulation and loss of sulphur from the cathodic electrode and passivation of the Li surface. In contrast, Figure 9(e–h) corresponds to the T-Li electrode after cycling under the same conditions. The surface displays the features of a protective coating still clearly discernible after cycling. The EDX elemental maps confirm the presence of sulphur; however, it is uniformly distributed in a lower amount (ca. 1.5 at%) compared to pristine Li. This result suggests that the protective layer on T-Li effectively limits polysulfide accumulation and mitigates parasitic reactions at the lithium surface, thereby contributing to improved interfacial stability and longer cycle life in Li//S cells.

## 4. Conclusions

In this study, a simple, cost-effective, and reproducible method has been developed to fabricate a protective interphase on lithium metal by immersing it in a nitrogen-saturated DOL/DME solution. The resulting T-Li surface exhibits enhanced interfacial stability and supports the formation of a stable and robust artificial SEI, contributing to improved electrochemical performance.

Comprehensive morphological and spectroscopic analyses (SEM, FTIR, XRD, and XPS) confirmed the presence of both organic and inorganic species but, surprisingly, no nitrogen-containing compounds were observed. Even if Li_3_N is expected to be present as a reaction product of Li and N_2_, it does not constitute a persistent component of the SEI. Instead, the complex reaction pathway, which involves reactive nitrogen species, leads to the formation of more stable species, such as LiOH and Li_2_O, as well as numerous organic compounds resulting from DOL degradation. Symmetric T-Li//T-Li cells demonstrated superior cycling stability over 500 cycles, reduced electrolyte decomposition, and a thicker and robust SEI even if compositionally similar to Li native layer.

When applied in full Li//S cells, the T-Li anode led to enhanced coulombic efficiency, higher specific capacity, improved capacity retention over prolonged cycling, and superior rate capability. These improvements are attributed to the protective layer’s ability to suppress polysulfide shuttling and maintain a stable interphase.

Overall, this approach represents a significant advancement towards the practical stabilization of lithium metal anodes for Li–S batteries. The method’s scalability and compatibility with ambient environments offer a promising pathway for large-scale implementation. Future work should aim to optimize the thickness and ionic conductivity of the protective layer to balance interfacial stability with efficient lithium-ion transport.

## Supplementary Material

Supplemental Material
