# Supplementary material for: Lithium interphase enhancement for applications in lithium–sulphur batteries
Source: Sci Technol Adv Mater. 2025 Nov 24;26(1):2593686. doi: 10.1080/14686996.2025.2593686 (PMC12707089; doi:10.1080/14686996.2025.2593686)
Supplement: Supplemental Material [file TSTA_A_2593686_SM7001.docx]

Supplementary material

Lithium interphase enhancement for applications in lithium-sulfur batteries

Antonio De Marco^aǂ^, Morteza Rahmanipour^a,#^, Gioele Pagot^b^, Giampaolo Lacarbonara^aǂ*^

^a^Department of Chemistry “Giacomo Ciamician”, University of Bologna, via Piero Gobetti 85, 40129 Bologna, Italy

^b^Section of Chemistry for Technology, Department of Industrial Engineering, University of Padova, via F. Marzolo 9, I-35131 Padova, Italy

*Corresponding Author: giampaol.lacarbonar2@unibo.it

ǂ These authors contributed equally to this work

# Present affiliation: PowerCo SE, Industriestraβe Nord, 38239 Salzgitter, Germany

**Imaging**


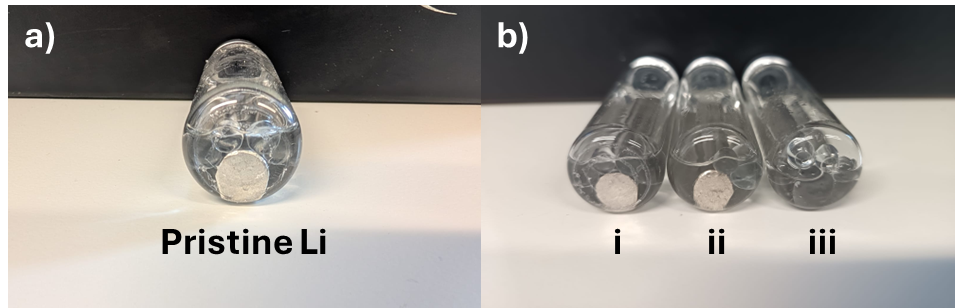


Figure S1. Pictures of (a) pristine Li, (b) i. Li left 2.5 h in DOL: DME, ii. Li left 2.5 h DOL: DME with Ar bubbling, iii. T-Li.


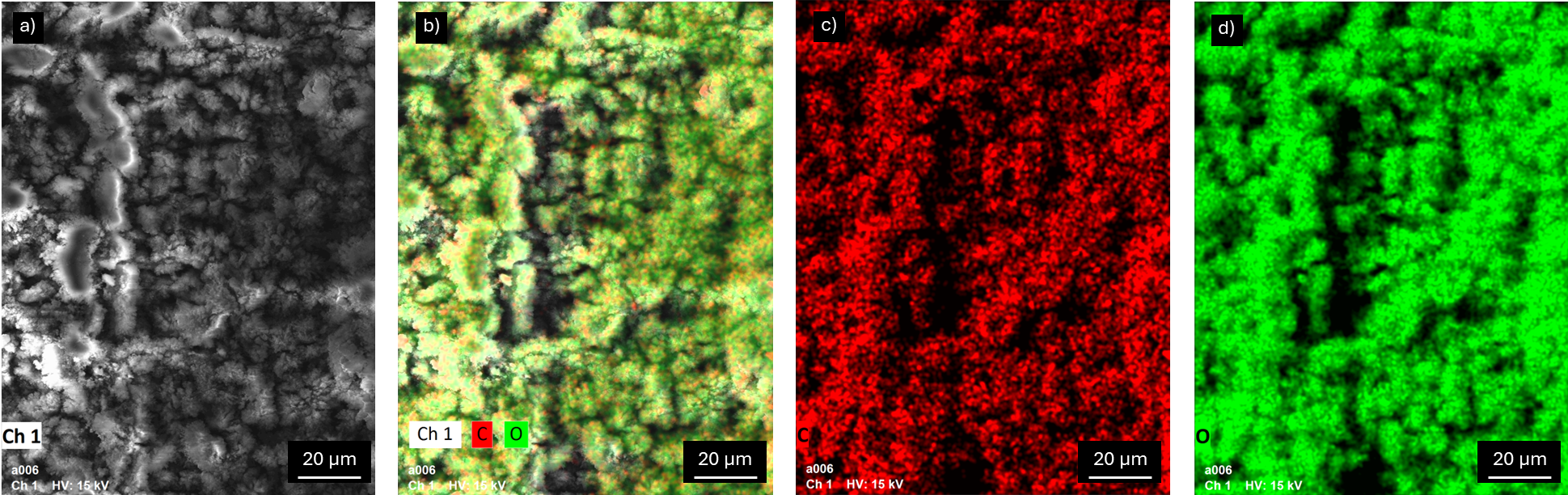


Figure S2. EDS images of T-Li sample.

**X-ray photoelectron spectroscopy**


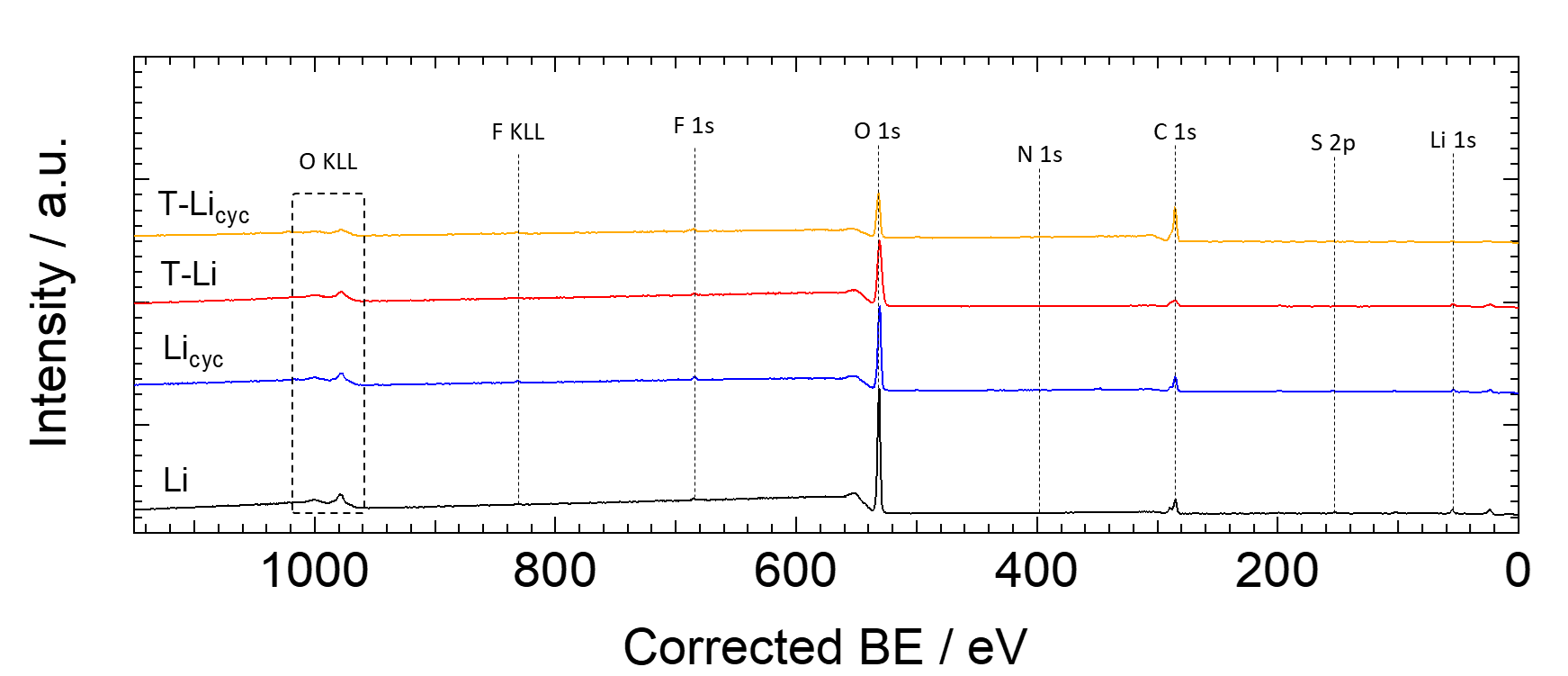


Figure S3. Survey XPS spectra and qualitative analysis of the surface elemental composition of the different samples.

Table S1. Surface elemental composition obtained from XPS studies.

| **Sample** | **C / at.%** | **O / at.%** | **Li / at.%** | **F / at.%** | **N / at.%** | **S / at.%** | **others / at.%** |
| --- | --- | --- | --- | --- | --- | --- | --- |
| Li | 15.81 | 40.91 | 40.42 | 1.33 | - | - | 1.53 |
| Li_cyc_ | 19.04 | 41.85 | 34.36 | 1.96 | 0.26 | 0.26 | 2.27 |
| T-Li | 13.65 | 39.05 | 45.83 | 0.86 | - | - | 0.61 |
| T-Li_cyc_ | 46.06 | 28.62 | 20.58 | 2.15 | 0.42 | 0.14 | 2.03 |

Table S2. Surface carbon relative composition obtained from XPS studies.

| **Sample** | **C-H / at.%** | **C-O/C-F / at.%** | **CO_3_^2-^ / at.%** |
| --- | --- | --- | --- |
| Li | 70.50 | 4.07 | 25.43 |
| Li_cyc_ | 65.37 | 13.94 | 20.69 |
| T-Li | 67.76 | 13.04 | 19.20 |
| T-Li_cyc_ | 71.72 | 12.45 | 15.83 |

Table S3. Surface oxygen relative composition obtained from XPS studies.

| **Sample** | **Li_2_O / at.%** | **LiOH / at.%** | **CO_3_^2-^ / at.%** | **ads / at.%** |
| --- | --- | --- | --- | --- |
| Li | - | 73.69 | 23.71 | 2.60 |
| Li_cyc_ | - | 72.85 | 20.10 | 7.05 |
| T-Li | 12.29 | 65.24 | 21.19 | 1.28 |
| T-Li_cyc_ | - | 52.53 | 39.30 | 8.16 |

Table S4. Surface lithium relative composition obtained from XPS studies.

| **Sample** | **LiOH/Li_2_O / at.%** | **Li_2_CO_3_ / at.%** |
| --- | --- | --- |
| Li | 76.39 | 23.61 |
| Li_cyc_ | 81.22 | 18.78 |
| T-Li | 100.00 | - |
| T-Li_cyc_ | 69.73 | 30.27 |

The ^1^H NMR spectrum recorded after dissolving the powder collected from the T-Li surface in DMSO-d_6_ is reported in Figure S4. The resulting spectrum reflects the soluble organic species formed on the lithium surface. A sharp singlet at ca. 8.5 ppm indicates the presence of aldehydic species, consistent with oxidative cleavage of the dioxolane ring. The intense, overlapping resonances between 3.1 and 3.7 ppm, partially covered by the residual water signal, are characteristic of protons α to oxygen atoms (–CH_2_–O–) and correspond to a mixture of DOL-derived fragments formed by polymerization. Minor features around 2.3–2.6 ppm suggest methylene groups adjacent to carbonyl functional groups (–CH_2_–CHO), while weaker aliphatic signals near 1.0–1.5 ppm can be attributed to trace dimethoxyethane (DME) or other low-molecular-weight by-products. Overall, the spectrum indicates that DOL underwent ring opening in the treatment conditions and subsequent oxidation, yielding a complex mixture of aldehydes, glycols, and ethers.

Figure S4. ^1^H NMR in DMSO-d_6_ of T-Li interphase obtained scratching the electrode after the treatment.

**Symmetric cell performance**


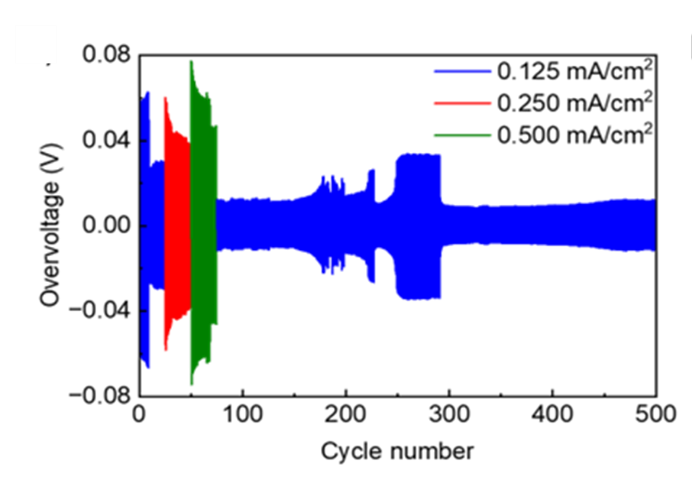


Figure S5. Voltage profiles in 1M LiTFSI DOL:DME with LiNO_3_ 0.45 M of Li//Li symmetric cell at different current density (0.5 h stripping/0.5 h plating).


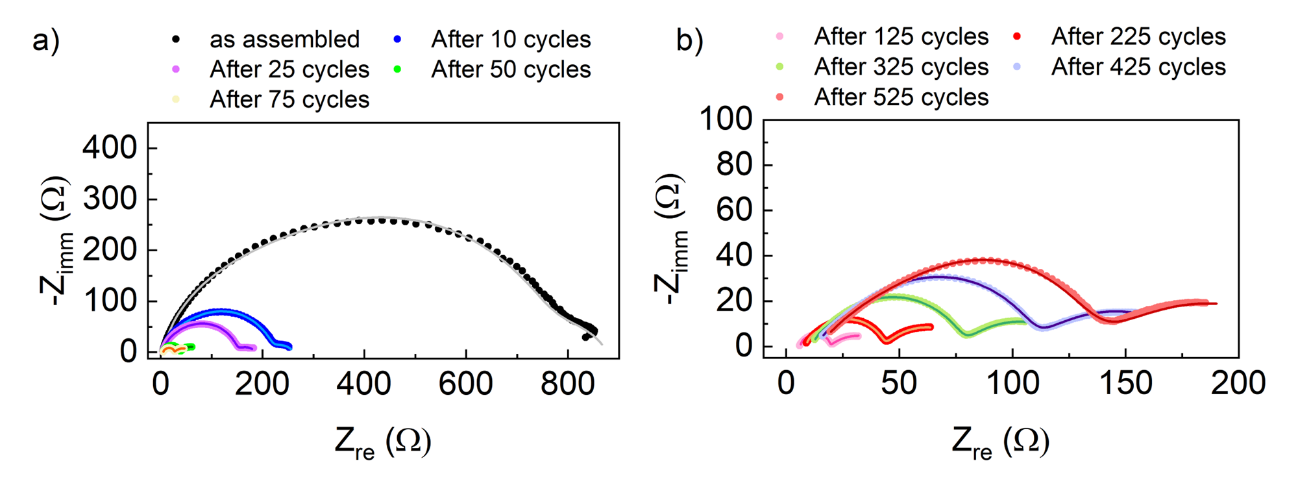


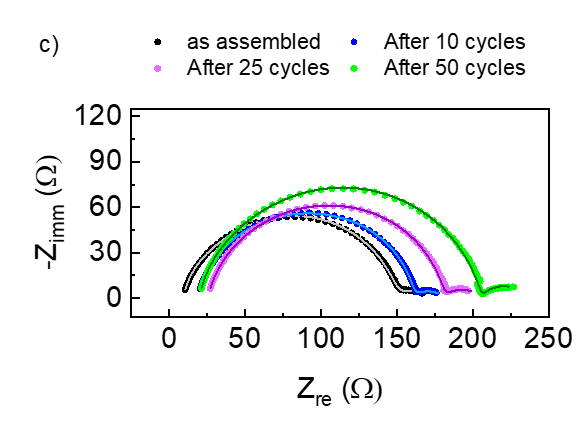


Figure S6. Impedance spectra and fitted curves for (a,b) T-Li//T-Li symmetric cells and (c) Li//Li cells at fresh state and after 10, 25, 50, 75, 125, 225, 325, 425, 525 cycles at 30°C. The solid lines are the data fittings.


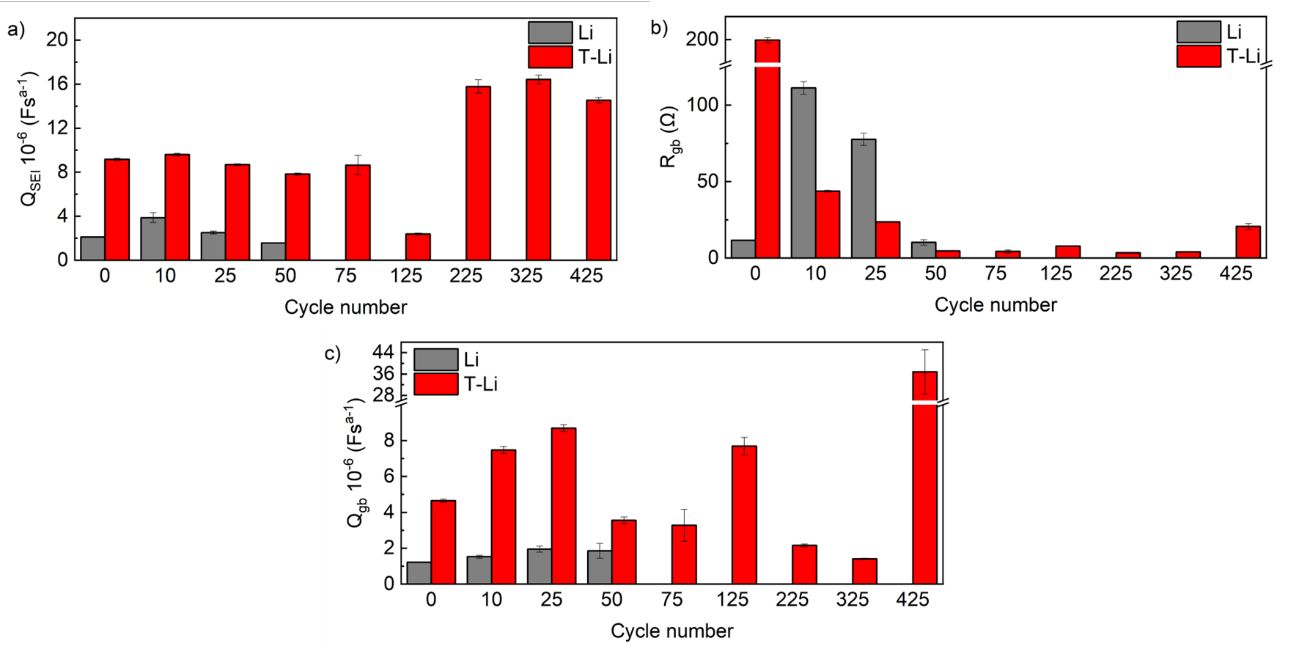


Figure S7. Evolution of Q_SEI_, R_gb_ and Q_gb_ over cycling for symmetric Li//Li and T-Li//T-Li cells.

Table S5. Results from the fitting of the EIS recorded for the T-Li//T-Li cell at 30°C.

| **Equivalent component** | **R_el_ Ω** | **R_SEI_  Ω** | **Q_SEI_ (10^-6^) F ∙ s^a-1^** | **a_SEI_** | **R_gb_  Ω** | **Q_gb_ (10^-6^) F ∙ s^a-1^** | **a_gb_** | **R_ct_ Ω** | **Q_dl_ (10^-2^) F ∙ s^a-1^** | **a_dl_** |
| --- | --- | --- | --- | --- | --- | --- | --- | --- | --- | --- |
| **As assembled** | 4.1 | 555 | 9.2 | 0.859 | 200 | 4.65 | 0.870 | 117 | 0.18 | 0.696 |
|  | ± 0.1 | ± 3 | ± 0.1 | ±0.001 | ±3 | ±0.09 | ± 0.002 | ± 5 | ± 0.02 | ±0.002 |
| **10 cycles** | 4.5 | 169 | 9.6 | 0.879 | 43.8 | 7.5 | 0.899 | 44.9 | 0.60 | 0.624 |
|  | ± 0.1 | ± 3 | ± 0.1 | ± 0.001 | ± 0.5 | ± 0.2 | ± 0.003 | ± 0.9 | ± 0.03 | ± 0.001 |
| **25 cycles** | 4.5 | 120.9 | 8.69 | 0.883 | 23.6 | 8.7 | 0.915 | 44.9 | 1.07 | 0.578 |
|  | ± 0.1 | ± 0.4 | ± 0.08 | ± 0.001 | ± 0.3 | ± 0.2 | ± 0.002 | ± 0.9 | ± 0.04 | ± 0.001 |
| **50 cycles** | 5.0 | 27.7 | 7.83 | 0.905 | 4.67 | 3.6 | 1 | 46.6 | 2.53 | 0.539 |
|  | ± 0.1 | ± 0.1 | ± 0.08 | ± 0.001 | ± 0.06 | ± 0.2 | ± 10^-6^ | ± 0.6 | ± 0.02 | ± 0.002 |
| **75 cycles** | 5.2 | 17 | 8.7 | 0.92 | 4 | 3.3 | 1 | 35.2 | 2.75 | 0.514 |
|  | ± 0.1 | ± 1 | ± 0.9 | ± 0.02 | ± 1 | ± 0.9 | ± 10^-6^ | ± 0.8 | ± 0.05 | ± 0.006 |
| **125 cycles** | 6.32 | 4.9 | 2.39 | 1 | 7.8 | 7.7 | 1 | 25.9 | 4.77 | 0.447 |
|  | ± 0.08 | ± 0.2 | ± 0.08 | ± 10^-6^ | ± 0.3 | ± 0.5 | ± 10^-6^ | ± 0.7 | ± 0.07 | ± 0.007 |
| **225 cycles** | 8.78 | 31.0 | 15.8 | 0.814 | 3.4 | 2.16 | 1 | 37.8 | 2.97 | 0.534 |
|  | ± 0.09 | ± 0.4 | ± 0.6 | ± 0.007 | ± 0.3 | ± 0.09 | ± 10^-6^ | ± 0.6 | ± 0.01 | ± 0.006 |
| **325 cycles** | 11.67 | 62.2 | 16.4 | 0.763 | 4.1 | 1.41 | 1 | 50 | 1.92 | 0.52 |
|  | ± 0.07 | ± 0.4 | ± 0.4 | ± 0.003 | ± 0.2 | ± 0.04 | ± 10^-6^ | ± 2 | ± 0.05 | ± 0.01 |
| **425 cycles** | 12.9 | 76 | 14.5 | 0.791 | 21 | 37 | 0.65 | 71 | 1.12 | 0.519 |
|  |  | ± 2 | ± 0.2 | ± 0.004 | ± 2 | ± 8 | ± 0.02 | ± 2 | ± 0.01 | ± 0.007 |
| **525 cycles** | 16.3 | 107 | 15.0 | 0.750 | 15 | 5 | 0.80 | 86 | 1.03 | 0.510 |
|  |  | ± 2 | ± 0.4 | ± 0.002 | ± 2 | ± 2 | ± 0.03 | ± 4 | ± 0.03 | ± 0.008 |

Table S6. Results from the fitting of the EIS recorded for the Li//Li cell at 30°C.

| **Equivalent component** | **R_el_ Ω** | **R_SEI_  Ω** | **Q_SEI_ (10^-6^)  F ∙ s^a-1^** | **a_SEI_** | **R_gb_  Ω** | **Q_gb_ (10^-6^)  F ∙ s^a-1^** | **a_gb_** | **R_ct_ Ω** | **Q_dl_  (10^-2^) F ∙ s^a-1^** | **a_dl_** |
| --- | --- | --- | --- | --- | --- | --- | --- | --- | --- | --- |
| **As assembled** | 9.22 | 127.7 | 2.109 | 0.866 | 11.6 | 1.22 | 1 | 22.8 | 0.138 | 0.479 |
|  | ± 0.06 | ± 0.2 | ± 0.004 | ± 0.001 | ± 0.1 | ± 0.02 | ± 10^-6^ | ± 0.2 | ± 0.002 | ± 0.002 |
| **10 cycles** | 18.5 | 31 | 3.9 | 0.993 | 111 | 1.53 | 0.861 | 21.3 | 0.188 | 0.495 |
|  | ± 0.2 | ± 4 | ± 0.4 | ± 0.006 | ± 4 | ± 0.08 | ± 0.005 | ± 0.4 | ± 0.007 | ± 0.002 |
| **25 cycles** | 25.3 | 77 | 2.5 | 0.914 | 77 | 2.0 | 0.860 | 26.7 | 2.2 | 0.491 |
|  | ± 0.2 | ± 4 | ± 0.1 | ± 0.005 | ± 4 | ± 0.2 | ± 0.007 | ± 0.7 | ± 0.09 | ± 0.003 |
| **50 cycles** | 19.7 | 175 | 1.57 | 0.863 | 10 | 1.9 | 0.990 | 30 | 3.11 | 0.618 |
|  | ± 0.2 | ± 2 | ± 0.04 | ± 0.004 | ± 2 | ± 0.4 | ± 0.009 | ± 3 | ± 0.02 | ± 0.002 |
